# Pre-exposure prophylaxis (PrEP) for MSM in low HIV incidence places: should high risk individuals be targeted?

**DOI:** 10.1038/s41598-018-30101-9

**Published:** 2018-08-03

**Authors:** Ngai Sze Wong, Tsz Ho Kwan, Owen T. Y. Tsang, Man Po Lee, Wing Cheong Yam, Wilson Lam, Wai Shing Leung, Jacky M. C. Chan, Kai Man Ho, Shui Shan Lee

**Affiliations:** 1 Stanley Ho Centre for Emerging Infectious Diseases, The Chinese University of Hong Kong, Hong Kong, China; 2 Jockey Club School of Public Health and Primary Care, The Chinese University of Hong Kong, Hong Kong, China; 3 Department of Medicine and Geriatrics, Princess Margaret Hospital, Hong Kong, China; 4 Department of Medicine, Queen Elizabeth Hospital, Hong Kong, China; 5 Department of Microbiology, The University of Hong Kong, Hong Kong, China

## Abstract

Pre-exposure prophylaxis (PrEP) targeting high-risk men who have sex with men (MSM) has been shown to be a cost-effective HIV control measure. However, the approach could be a challenge in low HIV incidence places with a low proportion of high-risk MSM. To examine the impact of PrEP in such setting in Asia, we developed an epidemic model and conducted cost-effectiveness analysis using empirical multicentre clinical and HIV sequence data from HIV-infected MSM in Hong Kong, in conjunction with behavioural data of local MSM. Without PrEP, the HIV incidence (per 100 person-years) would increase from 1.1 to 1.6 between 2011 and 2021. PrEP could avert 3–63% of total new infections in a five-year period (2017–2021), the variability of which depends on the implementation strategies and combination with test-and-treat. However, under current market drug price in 2016, the incremental cost per quality-adjusted life-year gained (QALYG) of PrEP (USD1583136/QALYG) is almost 3 times higher than test-and-treat intervention alone (USD396874/QALYG). Assuming 93% fall of PrEP drug price and in combination with test-and-treat, putting 30% of MSM on non-targeting PrEP would be more feasible, cost-effective (USD268915/QALYG), and could avert more new infections (40%). PrEP could contribute to HIV epidemic control in a low incidence place.

## Introduction

Despite a relatively high antiretroviral treatment (ART) coverage of over 80% in men who have sex with men (MSM) diagnosed with HIV infection since 2015, the annual number of new diagnoses has not fallen by more than 20%, as shown in some places like Australia and the United Kingdom (UK)^1,2^. In Australia, the proportion of HIV-infected MSM receiving treatment increased from 84% in 2014 to 88% in 2016, and the number of new diagnoses decreased by 8% per year^1^. In UK, the number of new diagnoses among MSM declined by 1% from 2014 to 2015, and the treatment coverage was 95% in 2015^2^. Besides treatment-as-prevention (TasP), the epidemic could further be controlled through addressing prevention needs of HIV negative individuals. The effectiveness of pre-exposure prophylaxis (PrEP), a biomedical preventive measure, was demonstrated in a number of studies, the results of which had been comprehensively reviewed^3^. In 2012, PrEP was approved by Food and Drug Administration (FDA). PrEP offers new opportunities to protecting HIV uninfected individuals including MSM, the HIV prevalence of which is often higher compared to other community groups in places like Europe and Asia Pacific^4,5^. PrEP as a preventive measure has been shown in modelling studies to avert a proportion of new infections and determined to be cost-effective in the estimations^6–9^. In a sub-study of iPrEX, a 99% transmission risk reduction was estimated under full adherence to PrEP usage^10^. The major challenge is to identify the most effective means for implementation.

In 2014, World Health Organization (WHO) recommended prioritizing populations at substantial risk of HIV infection when offering PrEP. WHO defined substantial risk based on local sub-population epidemiology (HIV incidence ≥3 per 100 person-years) in conjunction with personal assessment^11^. In low HIV incidence setting, introduction of PrEP has been shown in modelling studies (without consideration of cost) to contribute to epidemic control, as reported in Australia^6^, rural Zambia^7^, UK^12^, and Canada^13^. Some of these modelling studies and a cost-effectiveness analysis study in the Netherlands concluded with the recommendation of prioritizing high-risk groups in low incidence locality for PrEP^7,13,14^. Naturally, with the small proportion of high-risk MSM in these places, the cost of identifying and managing hidden population at high-risk of infection could be very high, while the actual proportion of MSM subsequently put on PrEP would be even smaller. In addition, stigma might be introduced by the targeting approach adopted for PrEP. High-risk MSM might be discouraged to take PrEP, while the programme cannot stop low-risk MSM from taking PrEP if they self-claim to be at high risk of HIV infection. Such phenomena could constitute new challenges to PrEP implementations.

We hypothesize that the additional benefit of targeting high-risk MSM in low incidence places for PrEP could be very small. To prove the hypothesis, epidemic modelling parameterized by clinical, HIV-1 sequences and behavioural data is a useful approach that has been applied in previous studies. To date, numerous PrEP modelling studies for MSM have been conducted in the United States, Canada, Europe, South America, Africa, and Australia, but only a few were reported in Asian countries (India and South Korea)^15–17^. Hong Kong, an Asian city, has a relatively stable HIV epidemic after an initial expansion phase predominantly affecting MSM, while the rate of infection in heterosexuals has remained low. The situation is different from India and South Korea, where heterosexual transmission continued to predominate. As of 2015, the cumulative number of reported HIV infected MSM cases was 3151 (41% of 7718 total cases) in Hong Kong^18^. Among MSM, the HIV prevalence is around 5%, and the annual number of new diagnoses had increased from 170 in 2010 to 464 in 2015^18^, despite the low incidence density of 1.1 per 100 person-year (http://www.chp.gov.hk/files/pdf/interim_statement_on_hiv_pre_exposure_prophylaxis.pdf). The rates of linkage to and retention in care, treatment initiation and viral load suppression are high (above 70%, http://www.aca.gov.hk/english/strategies/pdf/strategies17-21.pdf). In such setting, it is a challenge to effectively achieve further control of the HIV epidemic in MSM, and PrEP offers a new opportunity.

## Methods

### Study Population

Hong Kong is a metropolitan city, with a highly mobile population (58 million visitor arrivals in 2017, https://partnernet.hktb.com/en/home/index.html) and high population density (6777 persons/km^2^, https://www.bycensus2016.gov.hk/en/bc-mt.html). A previous study has estimated that 4.1% of adult male in Hong Kong were MSM, and 47.1% of them were sexually active^19^. The estimated number of sexually active MSM was around 50,000 in 2017. Apart from a pilot study, PrEP is currently not available as a public health preventive service in Hong Kong, and the cost of self-financed PrEP is very high (~USD23 per dose in 2016) in the absence of programmatic support. While some degree of PrEP awareness has been shown (26.6%), the current number of MSM on PrEP is speculatively small (1%)^20^.

### Data source

There are three HIV specialist clinics providing treatment to HIV-infected patients in the public service in Hong Kong. Clinical data (baseline and follow-up records) in 1985–2014, and HIV-1 genotype resistance testing (GRT) sequences in 1994–2012 of HIV-infected adult MSM were collected from all three clinics for model parameterization. The collected data accounted for 96% (1886/1958) of the cumulative reported HIV MSM cases by 2012. Results of our behavioural study on MSM were used to set behavioural parameter values^21^. Surveillance data were retrieved from the government’s annual surveillance reports^18^. We obtained institutional approvals from the Research Ethics Committee of the Joint Chinese University of Hong Kong – New Territories East Cluster, and Kowloon Central/Kowloon East Cluster of the Hospital Authority. Data access approval was granted by the Department of Health, Hong Kong Special Administrative Region Government, in compliance with the Personal Data (Privacy) Ordinance. Individual consent was waived. The datasets used in the current study are not publicly available because the data are owned separately by third parties. Access to these data and permission could be inquired through Department of Health, Hong Kong Special Administrative Region Government, Queen Elizabeth Hospital and Princess Margaret Hospital.

### Population delineation and mixing

We separated HIV-infected MSM into subgroups by analysing their HIV sequences^22^. Collected sequences (protease and partial reverse transcriptase of pol gene) were aligned by MUSCLE in MEGA6. From the constructed neighbour-joining tree, clusters were identified (bootstrap value ≥90, 1000 times simulation). They were further classified into subgroups by size of the clusters (number of cases) and by HIV subtype. Each subgroup was represented by one sub-model, and each sub-model included high and low-risk MSM for both HIV negative and positive MSM. A MSM is classified as belonging to the low-risk category if he has lower partner exchange rate (≤8 sexual partners per year), or high-risk if there has been higher partner exchange rate (Appendix A, p.1). Low-risk MSM were assumed to be in serial monogamy partnership while high-risk MSM were in random mixing partnership, corresponding with the low and high frequency of partner exchange in the model. Taking reference from our MSM behavioural study, 57% of MSM were deemed to be low-risk^21^. We assumed the presence of assortative mixing pattern, i.e. high-risk susceptible MSM mixed with high-risk infected MSM.

### Description of basecase model without PrEP

A deterministic compartmental model was developed in R for simulating HIV epidemic growth among MSM in Hong Kong in 1981–2021. The basecase model without PrEP included compartments as follow: (a) high-risk and low-risk susceptible MSM, (b) undiagnosed non-locally acquired infections, (c) undiagnosed individuals in acute infection, chronic infection categorized by CD4 levels of ≤200/μL, 201–350/μL, 351–500/μL, >500/μL, and AIDS, (d) diagnosed individuals in pre-treatment care with chronic infections and AIDS and (e) loss to follow-up before antiretroviral treatment (ART), (f) patients on treatment with non-suppressed (>500copies/mL) and suppressed viral load, and (g) loss to follow-up after treatment initiation. To incorporate the principles of TasP, we assumed minimal transmission risk for HIV-infected MSM with suppressed viral load (Appendix A, Table S2).

Details of model parameterization and model equations are described in Appendix A (p.1–6). Because of the lack of individual level data of non-locally acquired infections, we randomly distributed all annual reported non-local infections to each subgroup since the earliest seroconversion year of the cases in the subgroup (seroconversion year estimation methods described elsewhere^23^). The average difference in the number of new diagnoses in 2011 between basecase and 100 simulations was 4.9 persons (SD 3.12 persons) (simulation results in 1981–2021 in Appendix A, Supplementary Fig. S1). The model was fitted to the annual reported number of new MSM diagnoses in 1984–2011 by calibrating the proportion of low-risk HIV-infected MSM and sexual partner exchange rate of MSM in serial monogamy partnership in maximum likelihood estimation in R (Appendix A, Table S1).

### Description of model with PrEP

**Figure 1:**
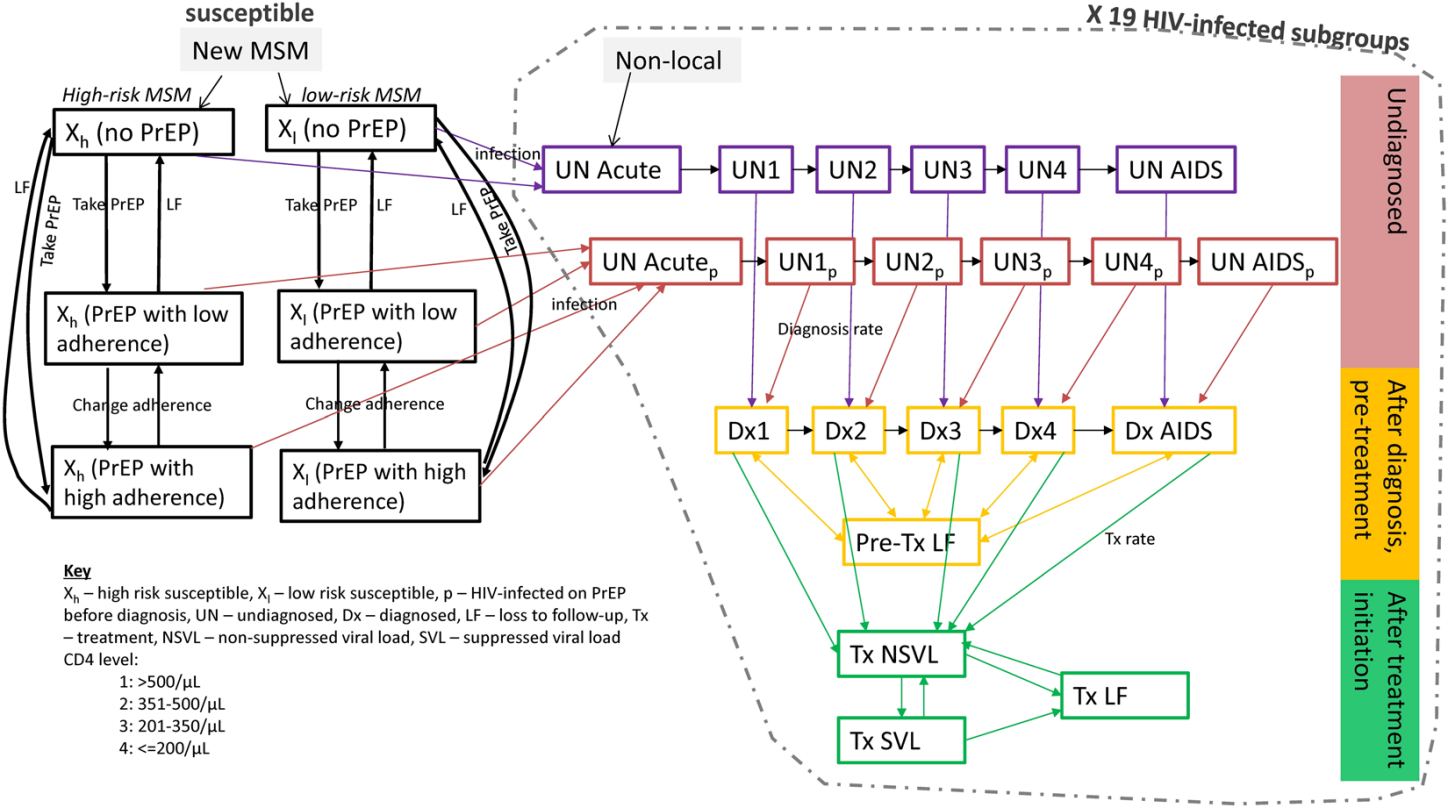
Flow diagram of basecase with PrEP model.

In the model, per-sex-act efficacy of PrEP was assumed to be 93%^24,25^, and effectiveness of PrEP was 70% in high adherence with ≥75% usage, and 23% in low adherence PrEP^15^. Based on basecase model, four more susceptible compartments were added to form a PrEP model, including the compartments of PrEP usage with high/low adherence stratified by high/low-risk MSM. (Fig. 1) PrEP users could drop out from PrEP usage, and change between high and low adherence in the model. There was however no change between high- and low-risk levels. To estimate the number of PrEP users infected with HIV, six undiagnosed compartments of acute infection, chronic infection by CD4 levels and AIDS were separately delineated for PrEP users.

### Cost and utility assumptions

For each HIV negative MSM on PrEP, high adherence was defined as 87.5% (mid-point of 75% and 100%) usage and low adherence as 38% (mid-point of 1% and 75%) usage of daily oral PrEP, attending 4 visits at HIV specialist clinics with HIV testing, a yearly test for creatinine, syphilis, *chlamydia trachomatis* (CT) and *Neisseria gonorrhoeae* (NG). HIV-infected MSM were assumed to have 4 specialist clinic visits, CD4 and viral load measurements in a year. For those on treatment, the ART cost reported locally was included. We adopted the CD4 level specific utility list applied in a previous cost-effectiveness analysis among MSM in Australia^26^. We assumed 3.5% annual discounted rate in the analysis^26^. List of cost and utility parameters is shown in Table S3, Appendix A.

### Scenarios development and sensitivity analyses

In the study, PrEP implementation was assumed to have started from 2017. We assumed that a proportion of susceptible MSM was on PrEP at the beginning of the year, with some dropping out in the remainder of the year. At the beginning of the following year, the total number of dropped-out users was replaced to maintain a constant coverage.

To compare the relative impacts of PrEP implementation with improving engagement in the cascade of HIV care, we developed scenarios in the model and conducted cost-effectiveness analysis from 2017 onwards, with different coverage of PrEP in (A) basecase, and with (B) a high rate of diagnosis and treatment initiation (minimum 90% from 2017, when test-and-treat was implemented):

- basecase without PrEP (A1); with 10%, 30% and 90% coverage of PrEP (A2) involving both low-risk and high-risk MSM (i.e. non-targeting approach) with low or high adherence usage; and involving high-risk MSM only (i.e. targeting approach) (A3) with low or high adherence usage;
- test-and-treat without PrEP (B1); with 10%, 30% and 90% coverage of PrEP through a non-targeting approach (B2) with low or high adherence usage; and through a targeting approach (B3) with low or high adherence usage;

As it was obvious that low adherence PrEP would not be cost-effective, only scenarios with high adherence PrEP had been developed in the cost-effectiveness analysis.

We performed two-way sensitivity analyses with different levels of PrEP coverage (0–95%, in 5% interval) in high- and low-risk MSM, stratified by high and low adherence in the epidemic model. We also developed hypothetical scenarios in high HIV incidence environment (≥3 per 100 person-years) to analyse the impact of PrEP implementation strategies. For cost-effectiveness analysis, we performed sensitivity analyses for different pricing scale of PrEP drug cost (year 2016 market price, generic price and zero cost).

### Disclaimer

The opinions and assertions contained herein are private views of the authors and do not necessarily reflect those of the affiliating institutions. Part of the preliminary model projection results have been presented at Health Research Symposium 2017 in Hong Kong, and at Asia Pacific AIDS & Co-infections Conference (APACC) 2018 in Hong Kong [oral presentations].

## Results

### Hong Kong’s cascade of HIV care in MSM

Among 1886 MSM cases with clinical data, the median age was 34 (IQR = 28–42), and 1541 (82%) were Chinese. For those with subtyping performed (1157 MSM), 254 (22%) belonged to subtype CRF01_AE, 835 (72%) to subtype B, and 68 (6%) to other subtypes. A total of 841 (45%) had a negative HIV test result before HIV diagnosis. Among them, 579 (69%) underwent the test ≤3 years before HIV diagnosis. By 2012, 1443 (77%) of MSM cases had initiated ART, after a median interval between HIV diagnoses and treatment initiation of 8 months (IQR = 2–29 months). Among those on treatment, 1286 (89%) cases had ever achieved viral load suppression (≤500/mL) by 2012 (details of temporal change of cascade of HIV care described elsewhere^27^).

### Population delineation

**Figure 2:**
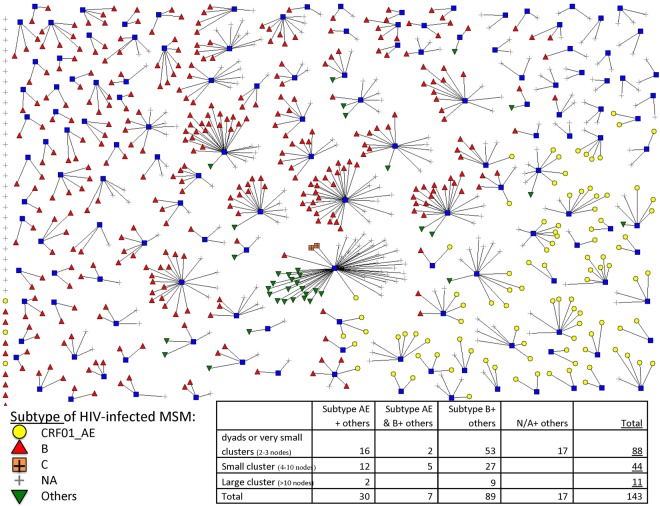
HIV sequences clusters drawn as social networks.

A total of 143 clusters were identified from 1135 HIV-1 sequences by phylogenetic analysis. There were 11 large clusters (>10 connected nodes), 44 small clusters (four to ten connected nodes), 88 dyads or very small clusters (two to three connected nodes) and 447 isolates. The distribution of these clusters by subtype is shown in Fig. 2. We categorized them into 19 MSM subgroups, with one subgroup for each large cluster (n = 11), and one subgroup for small clusters per subtype (n = 3), one subgroup for dyads or very small clusters per subtype (n = 4) and one subgroup for all isolates.

### Basecase model simulation without PrEP

**Figure 3:**
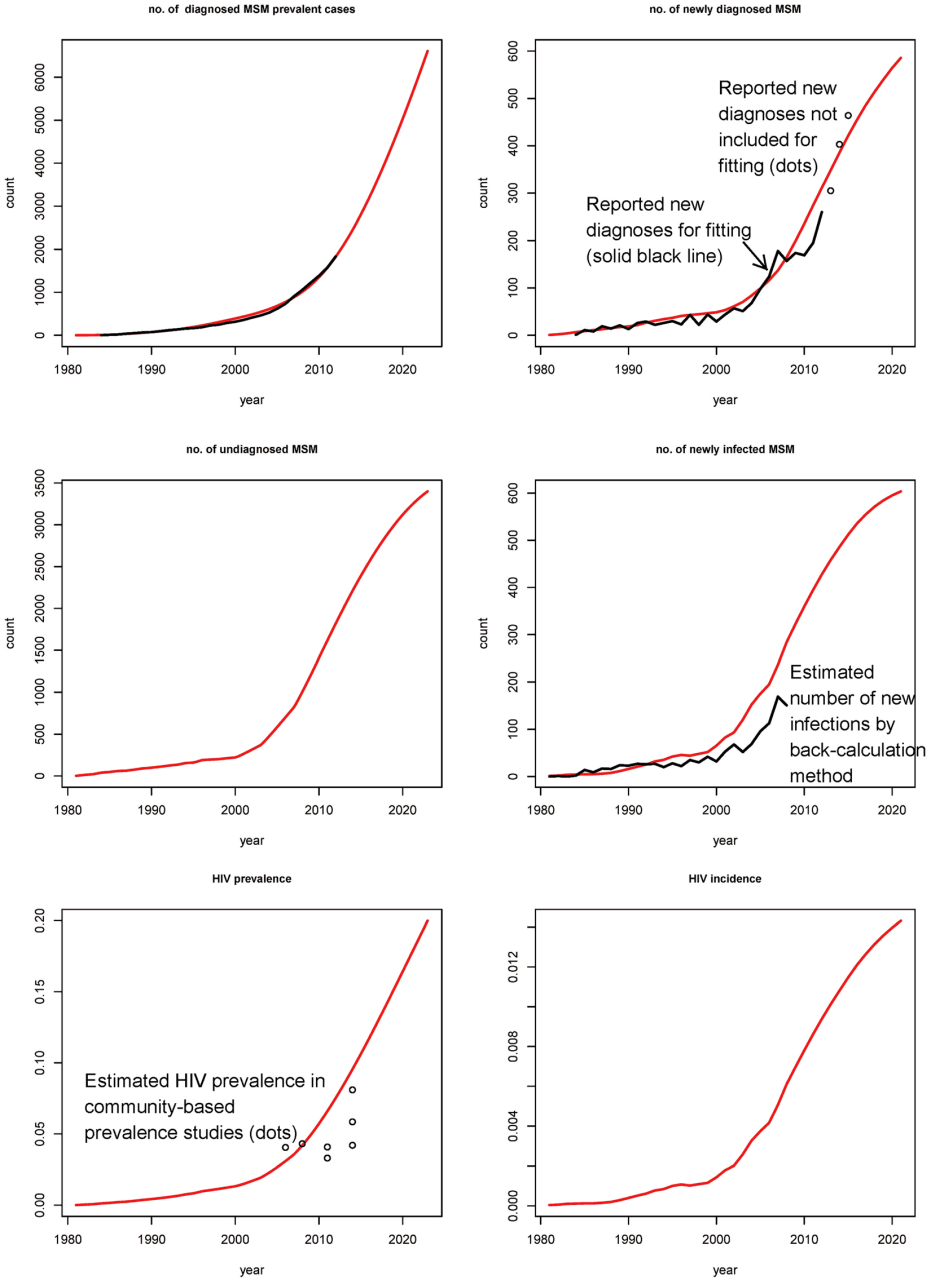
Model simulation results (red lines) in basecase scenario and observed data (black lines).

In the basecase model, the annual number of new diagnoses was projected to increase continuously from the year 2012 to 2021 (Fig. 3). Model estimates of HIV prevalence would increase from 0.08 in 2011 to 0.19 in 2021, while HIV incidence (per 100 person-years) would increase from 1.1 to 1.6. The number of locally acquired new infections would increase from 395 in 2011 to 604 in 2021. The estimated numbers in 1981–2011 were close to the annual reported number of new diagnoses and HIV prevalence as estimated from local community-based studies^18^.

### Impact projection of PrEP in basecase and the scenario of test-and-treat intervention

**Figure 4:**
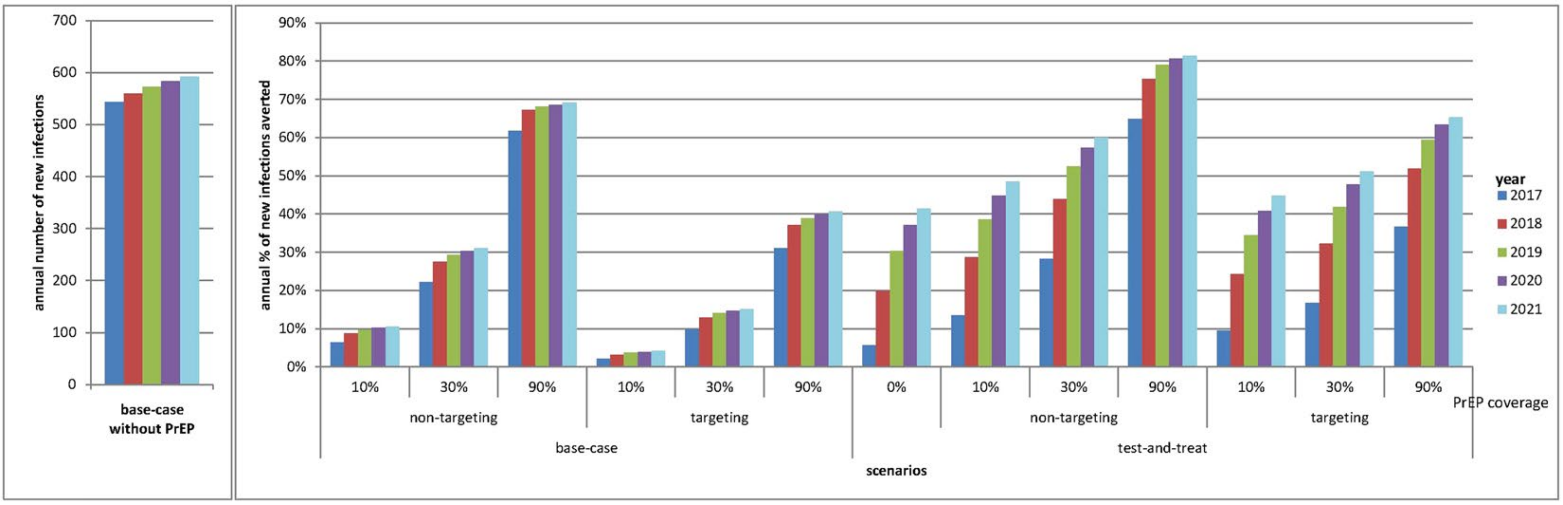
Projected annual proportion of new infections averted with different high adherence PrEP coverage (0%, 10%, 30%, 90%, targeting and non-targeting approaches) under (**a**) basecase scenario and (**b**) test-and-treat scenario in 2017–2021.

If PrEP is implemented from 2017, it could avert 8% (10% high adherence PrEP) to 55% (90% high adherence PrEP) of total new infections above basecase scenario in 2017–2021 (Table 1). The positive impact of low adherence PrEP would be smaller than high adherence under the same coverage (Appendix A, Supplementary Fig. S2). In the first year of PrEP implementation, the proportion of new infections averted would quickly reach the plateau in the second year (Fig. 4).

**Table 1:** Incremental cost-effectiveness of PrEP strategies over a 5-year time horizon.

	number of new infections	% of new infections averted	number of PrEP usage (person-year)	Discounted QALYG(1)	Plan A		Plan B		Plan C	
					Discounted incremental cost, USD (2)	(2)/(1)	(2)	(2)/(1)	(2)	(2)/(1)
**basecase**	3450	/	0							
non-targeting, 10%	2590	8%	17959	67	123458936	1842204	17294670	258064	9806914	146335
non-targeting, 30%	2048	23%	53910	212	370266861	1745524	51648582	243483	29176464	137545
non-targeting, 90%	942	55%	161936	526	1113780354	2115619	157156635	298518	89686050	170358
targeting, 10%	2754	3%	7629	24	52571166	2162072	7459389	306779	4277659	175926
targeting, 30%	2470	11%	22896	99	157200505	1583136	21831597	219862	12284040	123710
targeting, 90%	1780	31%	68752	287	472011282	1642874	65661580	228540	37001772	128788
**test-and-treat**	2075	23%	0	98	39055533	396874	39055533	396874	39055533	396874
non-targeting, 10%	1849	29%	17987	170	158411503	929215	52137608	305830	44642119	261863
non-targeting, 30%	1463	40%	53985	296	398568822	1345390	79665459	268915	57173233	192991
non-targeting, 90%	673	63%	162094	568	1127434311	1985645	170226003	299803	102714188	180901
targeting, 10%	1964	26%	7643	134	89432361	668940	44267840	331116	41082391	307290
targeting, 30%	1760	32%	22934	199	190572365	956132	55056540	276227	45498622	228274
targeting, 90%	1266	46%	68847	363	496573340	1366821	89865774	247356	61180726	168400

Plan A – market price for PrEP drug (annual cost of USD7880 at the end of 2017); Plan B – generic price for PrEP drug (annual cost of USD519); Plan C – zero cost for PrEP drug. All PrEP users are assumed to be in high adherence with an average of 87.5% usage per year. As we assume that 20% of high adherence users would change to low adherence users, whereas 10% of low adherence users would change to high adherence users in a year, a proportion of PrEP users would be in low adherence, with an average of 38% usage per year.

(2)/(1) = Discounted incremental cost-effectiveness (incremental $/QALYG).

Non-targeting – low-threshold approach with PrEP for both low- and high-risk MSM; targeting – PrEP for high-risk MSM only.

QALYG – quality-adjusted life-years gained.

In the scenario of test-and-treat without PrEP, the proportion of new infections averted would increase from 6% in 2017 to 41% in 2021 (Fig. 4). An additional 7–41% of total new infections would be averted in 2017–2021 if PrEP is implemented from 2017, ranging between coverage of 10% and 90% high adherence PrEP in the presence of a non-targeting approach (Table 1).

### Impact projection of PrEP implementation strategies – non-targeting approach vs targeting high-risk MSM approach

In basecase model, we compared between scenarios of different PrEP implementation strategies. Given the same PrEP coverage (10%, 30% and 90%) and with high adherence, a non-targeting approach could avert an additional 5–24% of the total new infections in five years (Table 1), or avert 4–31% more new infections yearly compared to a targeting approach in 2017–2021 (Fig. 4). Applying the test-and-treat model with PrEP, a non-targeting approach could avert an additional 3%, 9% and 17% of the new infections in five years compared to the targeting approach with 10%, 30% and 90% PrEP coverage, respectively (Table 1).

Taking cost and utility into account, PrEP would not be cost-effective with the current market drug price for PrEP (Plan A, Table 1), whereas test-and-treat without PrEP (USD396874/quality-adjusted life-year gained (QALYG)) was the most cost-effective intervention. Among the implementation strategies for PrEP, the minimum discounted incremental cost per QALYG was USD668940 (targeting 10% high risk MSM for PrEP with test-and-treat). If the drug cost for PrEP (USD7880/year, high adherence) drops to generic price (USD519/year, high adherence) and zero, the minimum discounted incremental cost per QALYG was USD 219862 and USD123710, respectively, with the strategy of targeting 30% high risk MSM for PrEP (Plan B and C, Table 1). With test-and-treat intervention, the minimum discounted incremental cost per QALYG for PrEP would be USD268915 (Plan B) and USD192991 (Plan C) with the non-targeting approach.

### Sensitivity analyses

From two-way sensitivity analyses, for every 5% increment in the number of high-risk MSM on high-adherence PrEP, the targeting approach would avert additional 3% new infections by 2021 (Appendix A, Supplementary Fig. S2). Beyond 30% coverage, the additional proportion of new infections averted would be reduced to 2%. Inclusion of low-risk MSM in the non-targeting approach would lead to more infections averted by 2021. On the other hand, with higher effectiveness of PrEP usage, the impact of PrEP would increase (Appendix A, Supplementary Fig. S3).

We developed a hypothetical scenario of high HIV incidence (≥3 per 100 person-years) to assess the situation in perspective. The impact is obviously high when targeting high-risk MSM for PrEP, as shown in the sensitivity analyses in Appendix A, Supplementary Fig. S4. When the coverage of PrEP (high-risk MSM in high adherence) is ≤20%, the extra proportion of new infections averted would be high. In the simulation, >7% reduction of new infections could be achieved per 10% expansion of PrEP coverage. However, the marginal benefit becomes diminished afterwards with further expansion of coverage.

## Discussion

In the deterministic compartmental model, we estimated that PrEP implementation since 2017 could avert 3–63% of total new MSM infections above basecase in Hong Kong in 2017–2021, the extent of which would vary with PrEP coverage and the effectiveness of implementing mixed interventions with the incorporation of test-and-treat intervention. Similar to other studies^7,13^, our results illustrated positive impact of PrEP intervention on HIV epidemic control among MSM, despite the low incidence in the locality.

An important finding of our study was that a non-targeting (low threshold for the recruitment of male satisfying the basic criteria of having unprotected sex with men, involving both high and low risk MSM) approach could avert more new infections than offering PrEP exclusively to MSM belonging to one distinct risk category. The finding was in line with those from UK’s modelling studies^12,28^. Although both studies suggested targeting MSM with higher sexual activity for PrEP, its definition was set at having more than one new sexual partner a year, with the adoption of a low threshold approach^12,28^. Our simulation results were however completely different from other PrEP modelling studies in low HIV incidence places that had adopted a targeted approach. Elsewhere, the results supported the prioritization of high-risk MSM for PrEP usage^8,13,14,29^. The discrepancy might have arisen from the different assumptions adopted for population mixing. Assortative mixing was assumed in our models, the results of which fitted well with the reported annual number of new diagnoses. We have developed a separate model with the incorporation of proportionate mixing pattern (Appendix A, Supplementary Fig. S5), which showed obvious overestimation. In addition, from our hypothetical model of a scenario with high HIV incidence, the relative impact of PrEP for high-risk MSM could avert more new infections than PrEP for any MSM. This was consistent with the findings in other modelling studies^8,13,14,29^.

In the implementation of PrEP, it is important to focus on adherence, as high adherence PrEP would avert additionally 10% or more new infections than low adherence (Appendix A), as shown in our results. Epidemiologically, our results confirmed that with PrEP, a mixed approach with implementation of interventions in both HIV negative and positive population could control the HIV epidemics effectively, as demonstrated in other modelling studies^12,30,31^. A modelling study in Los Angeles projected that 59% new infections could be averted by a mixed PrEP and test-and-treat strategy^30^, and a study in San Diego projected 50% reduction of new infections by the mixed approach^31^.

Taking the current high drug cost of PrEP (Plan A, annual USD7880/person in 2017) into consideration, PrEP intervention alone in the low HIV incidence setting does not appear to be cost-effective (at least USD1583136/QALYG). In the case of Hong Kong, a 93% reduction of the drug cost (Plan B, annual USD519/person in 2017) is desirable in order to demonstrate PrEP’s cost-effectiveness. Drug price as an important determinant of cost-effectiveness for PrEP is consistent with previous cost-effectiveness analyses in the published literature^32,33^. While targeting 30% high risk MSM for PrEP was 11% more cost-effective than non-targeting approach, the proportion of new infections averted would be 12% lower than that of non-targeting approach. Combined with test-and-treat intervention, a non-targeting approach with 30% coverage could avert 8% more new infections, and was 3% in Plan B or 15% in Plan C more cost-effective than targeting approach in the five-year period.

It is important to note that the cost implicated in PrEP includes not just drug cost but the accompanying programme cost of establishing/running an accessible PrEP service (cost not included in this study), coupled with the means to enrol eligible persons, the latter depending naturally also on the approach to be adopted. Resources should also be spent to enhance adherence. For a targeting approach, the identification of people at high risk of infection, who may otherwise be hidden in the low HIV incidence population, is a challenge. It can be argued that if the impact of PrEP implementation via a targeting or non-targeting approach is similar, it might not be necessary to pay for this additional cost aiming to identify MSM with very high risk behaviours. From a previous study, high-risk MSM had higher intention for future PrEP use^34^, suggesting that a non-targeting approach might already be able to attract more high-risk than low-risk MSM for PrEP. Similar relationship between intention to use PrEP and risk behaviour was observed in people who inject drugs^35^. Naturally, informing MSM and connecting them with an accessible PrEP service should be prioritized, which can normally be achieved with the collaborative efforts of community organisation and strategic use of Internet^36^.

This study carries some limitations. First, bisexual individuals were subsumed as MSM for analytic simplicity. Theoretically they could function as the epidemiological link, allowing HIV to spread from MSM to heterosexual population. In models projecting both heterosexual and MSM population, especially in the presence of a high proportion of bisexuals, further adjustment would be needed when applying our model structure. Second, the impact of condom usage on transmission risk was considered during high and low risk group classification, and as such, condom intervention and risk compensation (i.e. less condom use after PrEP usage) had not been modelled in the analyses. The potential impacts of sexually transmitted infections (STI) had not been factored in, though we acknowledged the potentially higher transmission risk of STI among PrEP users^37^. Third, only two types of sexual partnership, serial monogamy and random mixing were assumed, which might have underestimated the impact of concurrent partnership and group sex. We also assumed that the HIV transmission hazard of MSM was 1.2 times higher than heterosexuals. The value was determined under model calibration in our previous study^22^. As the basecase model estimation was close to the observed data (annual number of reported new diagnoses), model fitness was implied. Fourth, the effectiveness of PrEP was categorized in two levels, corresponding to low and high adherence of PrEP. However, with different mode of PrEP usage, including daily oral, event-driven and time-driven, the adherence pattern and overall effectiveness of PrEP would be very different in real life situation^10,25,38^. The effectiveness of PrEP would likely increase with the optimization of regimens in the future. From our sensitive analysis, more new infections would be averted with the increasing effectiveness of PrEP. Fifth, as the occurrence of adverse events of PrEP is unlikely to be related to the implementation approach, it had not been included in the model. However, we acknowledged the impact of adverse events in risk benefit calculation, when the HIV infection risk is minimal with PrEP usage. Finally, cost-effective analyses have been conducted to provide a broader perspective in evaluating possible strategic approach to PrEP. We have included drug cost and monitoring cost in the analyses, on the assumption that programmatic cost would be similar irrespective of the approach adopted. This can be an over-simplification of the actual situation should PrEP become implemented. Furthermore, the cost-effectiveness analyses only estimate a five-year period instead of longer duration. The incremental cost per QALYG might have been overestimated.

In conclusion, for low incidence places such as Hong Kong, PrEP could contribute to the control of the HIV epidemic in MSM in addition to the benefits from adopting a test-and-treat strategy. Offering PrEP without prioritization by behavioural risk in moderate coverage (say 30%) would be a feasible and cost-effective means for averting more new infections if PrEP drug cost is low enough. This strategy would likely be applicable for places with similarly low HIV incidence in particular in Asia, on the assumption that the networking patterns of MSM were similar. Our study shows that it is important not just to establish the baseline HIV incidence but consider the strategic approach for PrEP implementation in its development as part of a city/country’s HIV prevention programme.

## Electronic supplementary material

Appendix A
