# Supplementary material for: Pre-exposure prophylaxis (PrEP) for MSM in low HIV incidence places: should high risk individuals be targeted?
Source: Sci Rep. 2018 Aug 3;8:11641. doi: 10.1038/s41598-018-30101-9 (PMC6076226; doi:10.1038/s41598-018-30101-9)
Supplement: Supplementary file 1 — Appendix A [file 41598_2018_30101_MOESM1_ESM.pdf]

Appendix A

Pre-exposure prophylaxis (PrEP) for MSM in low HIV incidence places: should high risk individuals be targeted?

Ngai Sze WONG[1], Tsz Ho KWAN[2], Owen TY TSANG[3], Man Po LEE[4], Wing Cheong YAM[5], Wilson LAM[4], Wai Shing LEUNG[3], Jacky MC CHAN[3], Kai Man HO[3], Shui Shan LEE[1*]

1 Stanley Ho Centre for Emerging Infectious Diseases, The Chinese University of Hong Kong, Hong Kong, China

2 Jockey Club School of Public Health and Primary Care, The Chinese University of Hong Kong, Hong Kong, China

3 Department of Medicine and Geriatrics, Princess Margaret Hospital, Hong Kong, China

4 Department of Medicine, Queen Elizabeth Hospital, Hong Kong, China

5 Department of Microbiology, The University of Hong Kong, Hong Kong, China

Supplementary Material - additional model details

High- and low-risk groups

Susceptible (HIV negative) MSM were categorized into high and low-risk susceptible MSM. High-risk MSM have a higher partner exchange rate and low-risk MSM have a lower rate. Defining ≤8 sexual partners per year as low-risk, 57% of MSM were low-risk as observed in our ongoing MSM sexual behavioral study. Similarly, infected MSM were categorized into high-and low-risk infected MSM in each sub-model. The proportion of low-risk infected MSM in each sub-model adopted the parameter setting in our previous study with adjustment by calibration (Table S1).[1] In the model, low-risk MSM are assumed to be in serial monogamy while high-risk MSM are assumed to be in random mixing (very high rate of partner exchange) partnership. The annual rate of sexual partner exchange of low-risk infected varied between sub-models, in accordance to the size of clusters (Table S1). We did not assume the flow of infected MSM between sub-models. However, high-risk susceptible MSM could develop sexual partnership with high-risk infected MSM from any sub-models. Similarly, low-risk susceptible MSM could mix with low-risk infected MSM from any sub-models (i.e. assortative mixing pattern).

Table S1 proportion of low-risk infected MSM and characteristics of sexual partnership in MSM sub-models

Sub-models of different cluster types	Proportion of low-risk infected MSM	Annual rate of sexual partner exchange for low-risk MSM in serial monogamy
**isolates (1 node)**	79% + b	1 partner per 10 years
**dyads or very small clusters (2-3 connected nodes)**	70%+ b	a
**small clusters (4-10 connected nodes)**	66%+ b	a+1
**large clusters (>10 connected nodes)**		
**11-25 connected nodes**	55%+ b	a+2
**26-50 connected nodes**	50%+ b	a+2
**>50 connected nodes**	50%+ b	a+2

From model calibration, a= 6.7, b= -2%. The two parameters were used to adjust the value after the adoption from heterosexual model [1]

Sub-model (j = 1-19) equations in model with PrEP

We defined $\mathbf{S_h}$ as the number of high risk susceptible individuals, $\mathbf{S_l}$ as the number of low risk susceptible individuals, **A** as undiagnosed individuals in acute infection, **Un** as undiagnosed individuals in chronic infection, **UnAIDS** as undiagnosed individual with AIDS, **Dx** as diagnosed individuals, **DxL** as loss to follow-up before treatment initiation, **Tx** as patients on treatment, **TxL** as patients loss to follow-up after treatment initiation, **TxSVL** as patients on treatment with viral load suppression (<=500 copioes/mL), **TxNSVL** as patients on treatment with non-suppressed viral load (>500 copies/mL). We add "prep" in name of compartment to denote compartments with PrEP users only.

__**In the model:**__

Susceptible compartments without PrEP

$$\frac{dS_h}{dt} = -\sum_{j=1}^{19} \lambda_{h,j}[t] * S_h[t] + (1 - lowrisk) * aMSM[t] - \mathrm{PrEPh}[t] * \mathrm{S}_h[t] + dH * S_{hprep_H}[t] + dL * S_{hprep\_L}[t]$$

$$\frac{dS_l}{dt} = -\sum_{j=1}^{19} \lambda_{l,j}[t] * S_l[t] + (lowrisk) * aMSM[t] - \mathrm{PrEPl}[t] * \mathrm{S}_l[t] + dH * S_{lprep\_H}[t] + dL * S_{lprep\_L}[t]$$

Where **lowrisk** = proportion of MSM with ≤8 sex partners per year, **aMSM** = net change of MSM population, **PrEPh**=coverage of PrEP in high-risk MSM, **PrEPl**=coverage of PrEP in low-risk MSM;

Susceptible compartments with PrEP

Susceptible high-risk MSM on high adherence PrEP

$$\frac{dS_{hprep\_H}}{dt} = -\sum_{j=1}^{19} \lambda_{h,j}[t] * (1 - \mathrm{efficacyH}) * S_{hprep\_H}[t] + \mathrm{PrEPh}[t] * \mathrm{highadhH} * \mathrm{S}_h[t] - (dH + toLPrEP) * S_{hprep\_H}[t] + toHPrEP * S_{hprep\_L}[t]$$

Susceptible high-risk MSM on low adherence PrEP

$$\frac{dS_{hprep\_L}}{dt} = -\sum_{j=1}^{19} \lambda_{h,j}[t] * (1 - \mathrm{efficacyL}) * S_{hprep\_L}[t] + \mathrm{PrEPh}[t] * (1 - \mathrm{highadhH}) * \mathrm{S}_h[t] - (dL + toHPrEP) * S_{hprep\_L}[t] + toLPrEP * S_{hprep\_H}[t]$$

Susceptible low-risk MSM on high adherence PrEP

$$\frac{dS_{lprep\_H}}{dt} = -\sum_{j=1}^{19} \lambda_{l,j}[t] * (1 - \mathrm{efficacyH}) * S_{lprep\_H}[t] + \mathrm{PrEPl}[t] * \mathrm{highadhL} * S_l[t] - (dH + toLPrEP) * S_{lprep\_H}[t] + toHPrEP * S_{lprep\_L}[t]$$

Susceptible low-risk MSM on low adherence PrEP

$$\frac{dS_{lprep\_L}}{dt} = -\sum_{j=1}^{19} \lambda_{l,j}[t] * (1 - \mathrm{efficacyL}) * S_{lprep\_L}[t] + \mathrm{PrEPl}[t] * (1 - \mathrm{highadhL}) * S_l[t] - (dL + toHPrEP) * S_{lprep\_L}[t] + toLPrEP * S_{lprep\_H}[t]$$

Where **efficacyH**= efficacy of high adherence PrEP, **efficacyL**=efficacy of low adherence PrEP; **PrEPh**=coverage of PrEP in high-risk MSM, **PrEPl**=coverage of PrEP in low-risk MSM; **highadhH**=proportion of high-risk PrEP users in high adherence, **highadhL**=proportion of low-risk PrEP users in high adherence, **dH**=dropout rate of high adherence PrEP, **dL**=dropout rate of low adherence PrEP; **toLPrEP**=changing from high to low adherence PrEP; **toHPrEP**=changing from low to high adherence PrEP;

In each sub-model:

Undiagnosed compartments of non-PrEP users

$$\frac{dA_j}{dt} = \lambda_{l,j}[t]*S_l[t] + \lambda_{h,j}[t]*S_h[t] - (\alpha + mm)*A_j[t] + nonlocal_j[t]$$

$$\frac{dUn_j}{dt} = \alpha*A_j[t] - (vc_{i=1}[t] + ((\tau_{i=1} + mm)*(1 - vc_{i=1,j}[t])))*Un_{i=1,j}[t] + \sum_{i=2}^{4}[\tau_{i-1}*(1 - vc_{i-1,j}[t])*Un_{i-1,j}[t] - (vc_{i,j}[t] + ((\tau_i + mm)*(1 - vc_{i,j}[t])))*Un_{i,j}[t]]$$

$$\frac{dUnAIDS_j}{dt} = \tau_{i=4}*(1 - vc_{i=4,j}[t])*Un_{i=4,j}[t] - (vc_{i=5} + ((mm + mu[t])*(1 - vc_{i=5})))*UnAIDS_j[t]$$

Undiagnosed compartments of PrEP users

$$\frac{dAprep_j}{dt} = \lambda_{h,j}[t]*(1 - \text{efficacyH})*S_{hprep\_H}[t] + \lambda_{h,j}[t]*(1 - \text{efficacyL})*S_{hprep\_L}[t] + \lambda_{l,j}[t]*(1 - \text{efficacyH})*S_{lprep\_H}[t] + \lambda_{l,j}[t]*(1 - efficacyL)*S_{lprep\_L}[t] - (\alpha + mm)*Aprep_j[t]$$

$$\frac{dUnprep_j}{dt} = \alpha*Aprep_j[t] - (vc_{i=1}[t] + ((\tau_{i=1} + mm)*(1 - vc_{i=1,j}[t])))*Unprep_{i=1,j}[t] + \sum_{i=2}^{4}[\tau_{i-1}*(1 - vc_{i-1,j}[t])*Un_{i-1,j}[t] - (vc_{i,j}[t] + ((\tau_i + mm)*(1 - vc_{i,j}[t])))*Unprep_{i,j}[t]]$$

$$\frac{dUnAIDSprep_j}{dt} = \tau_{i=4}*(1 - vc_{i=4,j}[t])*Unprep_{i=4,j}[t] - (vc_{i=5} + ((mm + mu[t])*(1 - vc_{i=5})))*UnAIDSprep_j[t]$$

Diagnosed compartments

$$\frac{dDx_j}{dt} = vc_{i=1,j}[t]*Un_{i=1,j}[t] - (\tau_{i=1} + Tx_{i=1,j}[t] + dxlf_{i=1}[t] + mm)*Dx_{i=1,j}[t] + bc_{i=1}[t]*DxL_j[t] + \sum_{i=2}^{4}[vc_{i,j}[t]*Un_{i,j}[t] + \tau_{i-1}*Dx_{i-1,j}[t] - (\tau_i + Tx_{i,j}[t] trepgh - risk, l \text{ is low risk; "number is a ersons}\backslash\backslash\backslash\backslash M \text{ of any sub} -] + [vc_{i=5}*UnAIDS_j[t] + \tau_{i=4}*Dx_{i=4,j}[t] - (Tx_{i=5,j}[t] + dxlf_{i=5}[t] + mm + mu[t])*Dx_{i=5,j}[t] + bc_{i=5}[t]*DxL_j[t]]$$

$$\frac{dDxL_j}{dt} = \sum_{i=1}^{5} dxlf_i[t]*Dx_{i,j}[t] - \sum_{i=1}^{5} bc_i[t]*DxL_{i,j}[t] - mm*DxL_j[t]$$

$$\frac{dTxNSVL_j}{dt} = \sum_{i=1}^{5} Tx_{i,j}[t]*Dx_{i,j}[t] - (\omega_j + \kappa1_j[t] + mm)*TxNSVL_j[t] + \psi_j*TxSVL_j[t] + \gamma_j*TxL_j[t]$$

$$\frac{dTxSVL_j}{dt} = \omega_j*TxNSVL_j[t] - (\kappa2_j[t] + \psi_j + mm)*TxSVL_j[t]$$

$$\frac{dTxL_j}{dt} = \kappa1_j[t]*TxNSVL_j[t] + \kappa2_j[t]*TxSVL_j[t] - (\gamma_j + mm)*TxL_j[t]$$

Where **j**=infected MSM subgroups defined by phylogenetic results (group 1 to 19); **i** = 1 (CD4>500), 2 (CD4 351-500), 3 (CD4 201-350), 4 (CD4≤200), 5 (AIDS); **h** is high-risk, **l** is low risk; **t** is time, from 1981 to 2022, with time-step =1/52;

Force of infection λ

$\lambda_{l,j}$ = (lowl$_j$ *

$R_{l,x=1} * A_j[t] + R_{l,x=2} * \left(\sum_{i=1}^{4} Un_{i,j}[t] + \sum_{i=1}^{4} Unprep_{i,j}[t] + DxL_j[t] + \mathrm{TxL}_j[t]\right) + R_{l,x=3} * (UnAIDS_j[t] + UnAIDSprep_j[t]) + (R_{l,x=4} * \sum_{i=1}^{5} Dx_{i,j}[t]) + R_{l,x=5} * \mathrm{TxNSVL}_j[t] + R_{l,x=6} * \mathrm{TxSVL}_j[t]$

)/ N[t]

$\lambda_{h,j}$ = ((1-lowl$_j$)*

$R_{h,x=1} * A_j[t] + R_{h,x=2} * \left(\sum_{i=1}^{4} Un_{i,j}[t] + \sum_{i=1}^{4} Unprep_{i,j}[t] + DxL_j[t] + \mathrm{TxL}_j[t]\right) + R_{h,x=3} * (UnAIDS_j[t] + UnAIDSprep_j[t]) + (R_{h,x=4} * \sum_{i=1}^{5} Dx_{i,j}[t]) + R_{h,x=5} * \mathrm{TxNSVL}_j[t] + R_{h,x=6} * \mathrm{TxSVL}_j[t]$

)/ N[t]

Where **lowI$_j$** is the proportion of low risk infected MSM; **N** is the total number of MSM in the community regardless of HIV serostatus
N[t] = mN*msmp + aMSM[t]
Where **mN** is the number of adult male population (15-64 years old) in 1981; **msmp** is the proportion of sexual active MSM in adult male population, assuming constant over time; **aMSM** is the annual net change of number of MSM, which is proportional to the net change of adult male population size
and

reproduction number divided by duration for low risk level: $R_{l,x} = \frac{\beta_x * c_l * \mathrm{duration}_x}{(\beta_x + c_l + \frac{1}{duration_x})} \div \mathrm{duration}_x$

reproduction number divided by duration for the high risk level: $R_{h,x} = \frac{\beta_x * \mathrm{duration}_x}{\mathrm{duration}_x} = \beta_x$

Where **x** = 1 (acute infection), 2 (undiagnosed chronic infection or loss to follow-up), 3 (undiagnosed AIDS), 4 (diagnosed chronic infection and AIDS), 5 (treatment without viral load suppression), 6 (treatment with viral load suppression), **β**= transmission hazard, **c** = number of sex partners per year, **duration**= period of each stage (1/α for acute infection, 1/τ for chronic infection)

Table S2 Model parameters

**Description**	**Symbol**	**Estimated value**	**Further explanation / Source**
**Biological parameters**			
Transmission hazard in heterosexuals (per year)	β		
Undiagnosed acute infection	$\beta 1$	2.76	[2]
Undiagnosed chronic infection	$\beta 2$	0.106	[2]
Undiagnosed AIDS	$\beta 3$	0.76	[2]
Diagnosed chronic infection and AIDS	$\beta 4$	0.036	[2]
Initiated treatment with NSVL	$\beta 5$	0.179	[3]
Initiated treatment with SVL	$\beta 6$	0.00178	[3]
Lost to follow-up	$\beta 2$	0.106	[2]
Adjusting vector for estimating transmission hazard in MSM		1.2	Calibration
Duration of disease stages			
Acute infection	$1/\alpha$	0.5 years	[2]
Chronic infection	$1/\tau$	Total 6.5 years	Clinical data
CD4 >500/μL	$1/\tau_{i=1}$	22 months	Clinical data
CD4 351-500/μL	$1/\tau_{i=2}$	21 months	Clinical data
CD4 201-350/μL	$1/\tau_{i=3}$	19 months	Clinical data
CD4 ≤200/μL	$1/\tau_{i=4}$	16 months	Clinical data
Effectiveness of PrEP usage			
High adherence	efficacyH	70%	[4]
Low adherence	efficacyL	23%	[4]
Annual mortality rate for AIDS	mu		
Before 2000		0.3	Clinical data
on or after 2000		0.1	Clinical data
Mortality rate for general population	mm	0.00517	Population census
**Behavioral parameters**			
% of low risk group	lowrisk	57%	Data from our ongoing study
Duration of stable sexual partnership	c		Calibration
**PrEP usage**			
Proportion of high risk group on PrEP	PrEPh	[tested in sensitivity analysis]	Assumption
High adherence	highadhH	[tested in sensitivity analysis]	Assumption
Low adherence	1-highadhH	1- high adherence proportion	Assumption
Proportion of low risk group on PrEP	PrEPl	[tested in sensitivity analysis]	Assumption
High adherence	highadhL	[tested in sensitivity analysis]	Assumption
Low adherence	1-highadhL	1- high adherence proportion	Assumption
Drop-out rate of PrEP usage	dH, dL	20% for both high and low adherence	

Annual rate of changing PrEP adherence			
From high to low adherence	toLPrEP	20%	[4]
From low to high adherence	toHPrEP	10%	[4]
**Cascade of HIV care**			
Diagnosis rate	vc_i	Varied across time and sub-groups	Clinical data
Loss to follow-up rate (before treatment)	$dxlf_i$	Varied across time and sub-groups	Clinical data
Back to care rate (before treatment)	bc_i		
Treatment initiation rate	Tx_i	Varied across time and sub-groups	Clinical data
Treatment loss to follow-up rate	κ1, κ2	Varied across time and sub-groups	Clinical data
Back to treatment rate	γ	Varied across time and sub-groups	Clinical data
Viral suppression rate	ω	Varied across sub-groups	Clinical data
Viral rebound rate	ψ	Varied across sub-groups	Clinical data
**Others**			
Non-locally acquired infections	nonlocal	Varied across time and sub-groups	Annual surveillance reports for the total number of non-local infections,[5] and randomly assigned to subgroups, validated by simulations
Male (aged 15-64) population size	mN	1904357 in 1981	[6]
Proportion of sexually active MSM in adult male population	msmp	0.02	[7]
Annual net change of number of MSM	aMSM	Net change of mN * msmp, change over time	[6], [7]

Supplementary figures

Supplementary Fig. S1. Model simulations (100 times) of annual number of new diagnoses by randomly assigning non-locally acquired infections to 19 sub-models

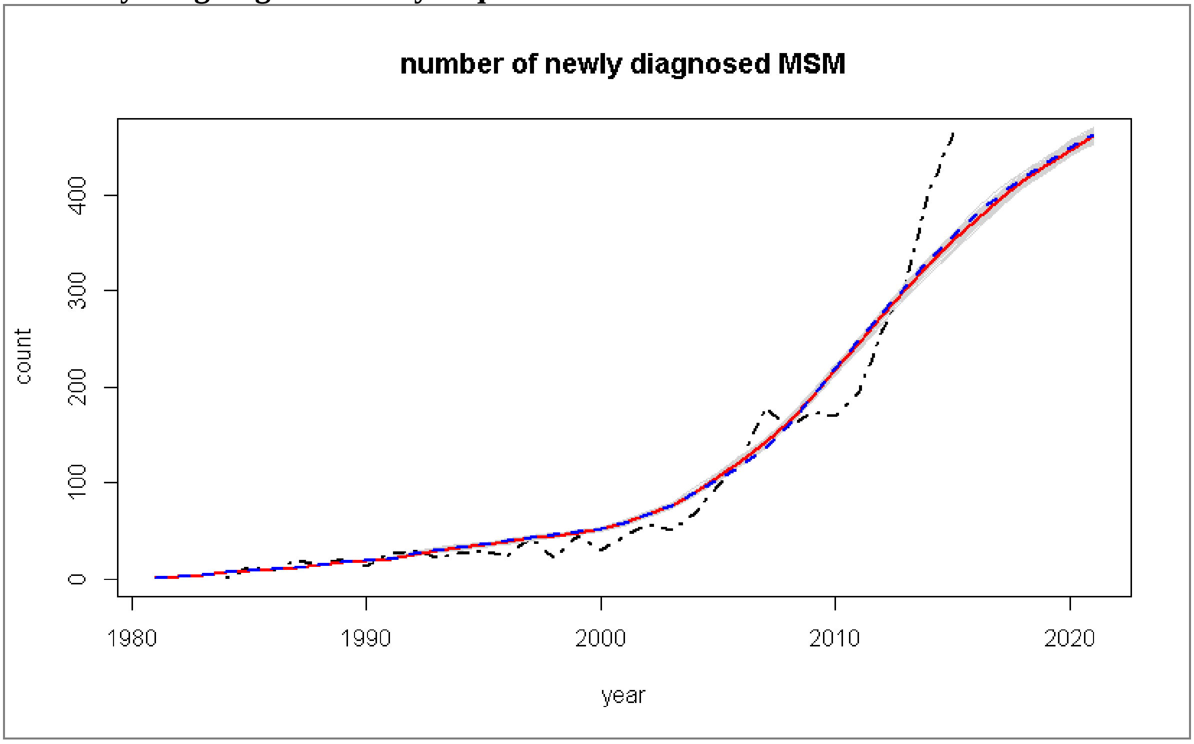

Supplementary Fig. S2. Estimates of proportion of new infections averted in 2021 above basecase scenario without PrEP in sensitivity analyses of 0-95% low adherence and high adherence PrEP coverage target high and low-risk groups

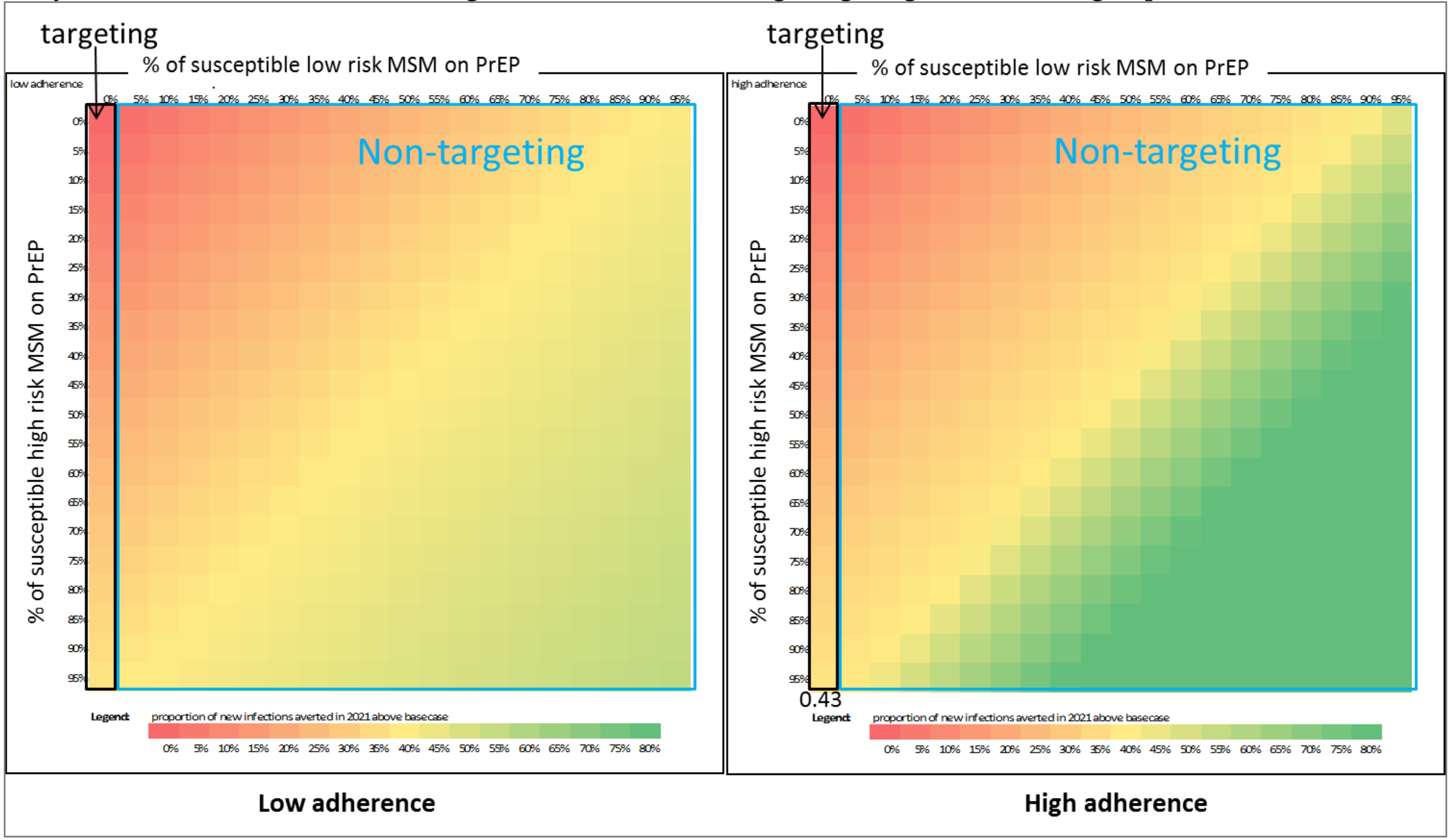

Supplementary Fig. S3. Impact of overall effectiveness of PrEP along high adherence PrEP coverage (1%, 10% and 90%) in MSM

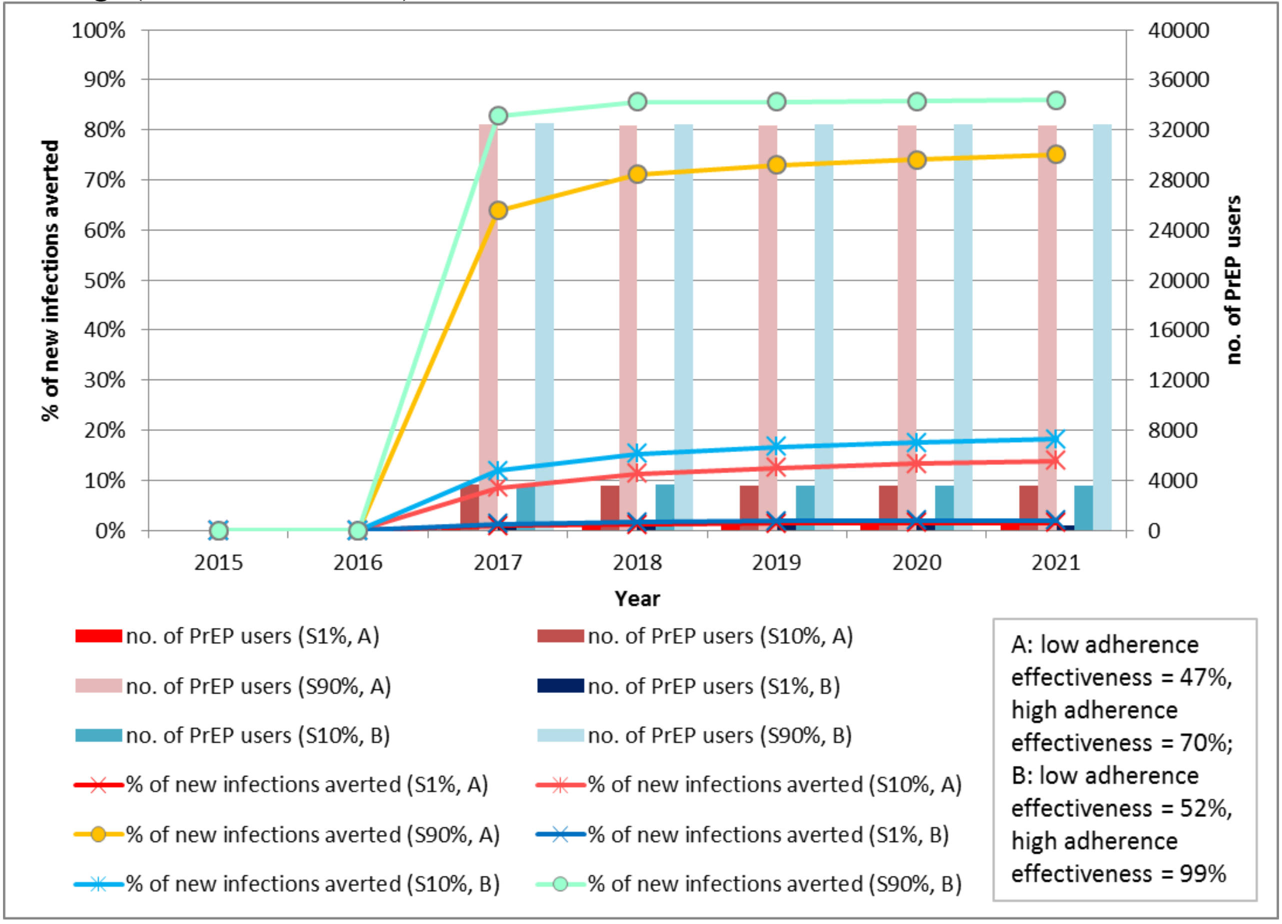

The impact of PrEP effectiveness were analysed in scenarios of increasing the effectiveness of PrEP (A) from 23% to 47% in low adherence, and effectiveness of high adherence remains 70%; (B) increasing from 23% to 52% in low adherence, and from 70% to 99% in high adherence.

Supplementary Fig. S4. In high HIV incidence place, estimates of proportion of new infections averted in 2021 above scenario without PrEP in sensitivity analyses of 0-90% high adherence PrEP coverage target high and low-risk groups

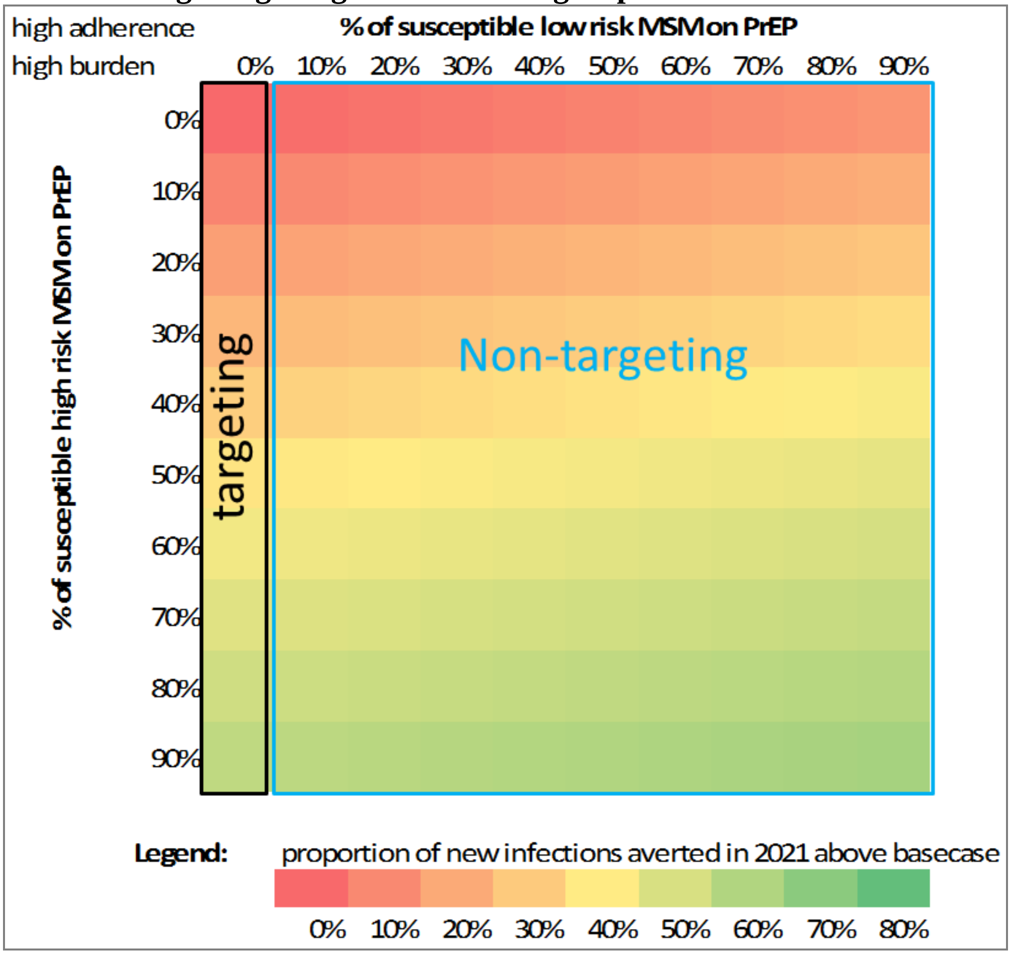

Supplementary Fig. S5. Model simulation results by random mixing (red lines) and assortative mixing in 19-model (blue lines), in comparison with observed data (black dots)

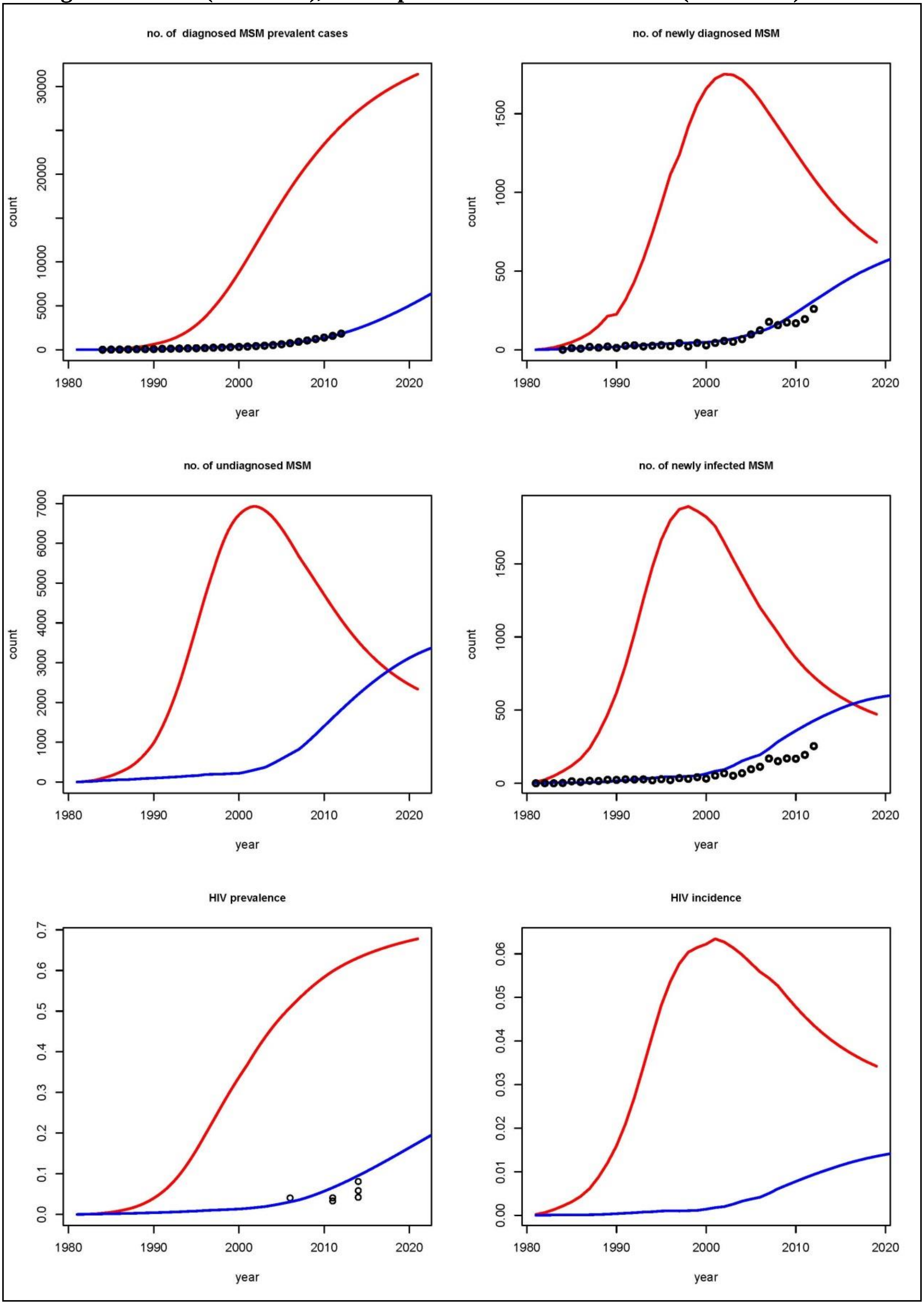

Table S3 parameters for cost-effectiveness analysis

**Parameter**	**Value**	**Source**
**Annual cost in year 2016 (USD)**		
Specialist visit (4 visits per year for HIV patients and PrEP users)	$536	Specialist visit cost as listed in http://gia.info.gov.hk/general/201106/29/P201106290186_0186_80935.pdf ; Further adjustment was made by inflation rate (composite consumer price index) estimated by Census and Statistics Department, Hong Kong Special Administrative Region.
PrEP annual cost (high adherence in 87.5% usage, daily oral HKD188 (~$24) per dose)	$7703	Market price of PrEP drug in 2016; With reference to the previous study,[4] high adherence PrEP users were defined as ≥75% usage. We take the mid-point of 75% and 100% for the estimation of PrEP annual cost; Further adjustment was made by inflation rate (composite consumer price index) estimated by Census and Statistics Department, Hong Kong Special Administrative Region.
PrEP annual cost (low adherence in 38% usage, daily oral HKD188 (~$24) per dose)	$3345	Market price of PrEP drug in 2016; We take the mid-point of 1% and 75% for the estimation of low adherence usage of PrEP (38%).[4]; Further adjustment was made by inflation rate (composite consumer price index) estimated by Census and Statistics Department, Hong Kong Special Administrative Region.
Testing cost for PrEP (HIV per visit, and creatinine, syphilis, CT, NG once per year)	$104	
ART annual cost for HIV-infected	$16761	
cost for CD4 and viral load measurement (4 times per year)	$410	
**Utility**		
Noninfected	1	[8]
Pre-treatment with		
CD4 >=350	0.935	[8]
CD4 200-349	0.818	[8]
CD4 <200 or AIDS	0.702	[8]
On treatment with NSVL	0.818	As viral load level is significantly associated with CD4 level, patients with NSVL are commonly with lower CD4 level and probably lower utility.
On treatment with SVL	0.935	We assume that CD4 level of patients with SVL is recovering to a satisfactory level with high utility.
Loss to follow-up	0.818	Patients lost to follow-up are mostly with NSVL.
Death	0	
**Annual discounted rate**	3.5%	[8]

NSVL – non-suppressed viral load (>500 copies/mL); SVL – suppressed viral load (≤500 copies/mL)

Reference

1. Wong, N.S. *The incorporation of viral load measures at sub-population level for modelling the HIV epidemic in Hong Kong*. (2015).
2. Hollingsworth, T.D., Anderson, R.M. & Fraser, C. HIV-1 transmission, by stage of infection. *J Infect Dis.* **198**, 687-93 (2008).
3. Jean, K. et al. Effect of early antiretroviral therapy on sexual behaviors and HIV-1 transmission risk among adults with diverse heterosexual partnership statuses in Côte d'Ivoire. *The Journal of Infectious Diseases*.**209**,431-40 (2014).
4. Mitchell, K.M. et al. Potential impact of pre-exposure prophylaxis for female sex workers and men who have sex with men in Bangalore, India: a mathematical modelling study. *J Int AIDS Soc*. **19,** 20942 (2016).
5. Virtual AIDS Office of Hong Kong. *Virtual AIDS Office – HIV surveillance office*. http://www.info.gov.hk/aids/english/surveillance/sur_report/hiv15.pdf (2015)
6. Census and Statistics Department HKSAR. *Demographic trends in Hong Kong 1981–2011*. http://www.censtatd.gov.hk/hkstat/sub/sp150.jsp?productCode=B1120017 (2012)
7. Lau, J.T., Siah, P.C. & Tsui, H.Y. A study of the STD/AIDS related attitudes and behaviors of men who have sex with men in Hong Kong. *Arch Sex Behav*. **31**, 367-73 (2002).
8. Schneider, K., Gray, R.T. & Wilson, D.P. A Cost-effectiveness Analysis of HIV Preexposure Prophylaxis for Men Who Have Sex With Men in Australia. *Clin Infect Dis*. **58**, 1027-34 (2014).
